# ﻿*Primulayanbianensis* (Primulaceae), a new species in ﻿*Primula* sect. ﻿*Cortusoides* from Sichuan, China

**DOI:** 10.3897/phytokeys.252.140026

**Published:** 2025-02-14

**Authors:** Tian Shuai, Hong-qiang Lin, Lei Cai, Yu-fan Chen, Zhi-kun Wu

**Affiliations:** 1 Department of Pharmacy, Guizhou University of Traditional Chinese Medicine, Guiyang, 550025, Guizhou, China Guizhou University of Traditional Chinese Medicine Guiyang China; 2 Sichuan Wolong National Natural Reserve Administration Bureau, Wenchuan, 623006, Sichuan, China Sichuan Wolong National Natural Reserve Administration Bureau Sichuan China; 3 Yunnan Key Laboratory for Integrative Conservation of Plant Species with Extremely Small Populations, Kunming Institute of Botany, Chinese Academy of Sciences, Kunming, 650201, Yunnan, China Kunming Institute of Botany, Chinese Academy of Sciences Kunming China

**Keywords:** New species, *Primula* sect. *Cortusoides*, Sichuan, yan bian bao chun

## Abstract

*Primulayanbianensis* T.Shuai, Lei Cai & Z.K.Wu, a new species of Primulaceae from Sichuan, China, is described and illustrated. Morphological evidence supports *P.yanbianensis* as a member of P.sect.Cortusoides, which is characterised by lack of farina, generally well furnished with multicellular hairs, subrounded leaf blades are more or less lobed, with distinct petiole and a deeply cordate base and campanulate or narrowly campanulate calyx not accrescent after anthesis. The new species is characterised by having umbel in whorls of 1–2, scape usually lower than leaf clusters, bracts broadly ovate and rose to pink petals distinctly veined. The distribution, morphological comparison with closely-related species and conservation status of the new species are also provided.

## ﻿Introduction

*Primula* L. is one of the largest genera in Primulaceae, encompassing ca. 545 species worldwide (POWO 2024). Most *Primula* species are native to temperate and alpine regions of the Northern Hemisphere, with only a few species occurring in the Southern Hemisphere (Hu 1990; Hu 1994; Richards 2002). The south-western region of China (especially the Himalayan-Hengduan Mountains) is the centre of diversity of the genus, with more than 300 species recorded, which are distributed mainly in western Sichuan, eastern Xizang and north-western Yunnan (Hu 1990; Hu and Kelso 1996; Richards 2002).

PrimulaSect.Cortusoides Balf. f. (39: 140, 1913) (Balfour 1913), comprising about 25 species worldwide and 19 species being recorded in China (of which *P.pelargoniifolia* G.Hao, C.M.Hu & Z.Y.Liu and *P.longipilosa* Ze H.Wang & H.Peng are two newly-described species in recent years (Xu et al. 2014a; Wang et al. 2022), is widely distributed in central and eastern Asia, but its distributional centre is in SW China (Hu 1990; Xu et al. 2014a; Wang et al. 2022). Morphologically, the section is characterised by the plants being efarinose, but generally well furnished with multicellular hairs, subrounded leaf blades are more or less lobed, with distinct petiole and a deeply cordate base and campanulate or narrowly campanulate calyx not accrescent after anthesis (Smith and Fletcher 1944).

As one of the hotspots of biodiversity in China, Sichuan has over ca. 170 species of *Primula* distributed all over its range (Wu 1999). With the increased exploration of the region, many new additions of *Primula* species have constantly been reported in Sichuan over the past two decades (Hu and Ceng 2003; Hu and Hao 2006; Wu et al. 2013; Xu et al. 2014b, 2015, 2016a, 2016b, 2016c, 2017a, 2017b, 2019; Ju et al. 2018, 2021, 2023; Yuan et al. 2018; Li et al. 2023; Luo et al. 2023).

During a botanical expedition to Yanbian, Sichuan, south-western China in June 2017, we discovered a peculiar *Primula* population with long white multicellular hairs all over the plant, subrounded leaf blades being more or less lobed, with distinct petiole and a deeply cordate base and narrowly campanulate calyx. These features indicate that it should be a member of the P.Sect.Cortusoides. However, its scape is shorter than the leaves, umbel is in whorls of 1–2, bracts are broadly ovate and rose to pink petals are distinctly veined, all of which indicate it to be a distinct species. We surveyed the site and adjacent area again and collected specimens in July 2020 and June 2024. After a full observation of the morphological characteristics and comparing the relevant literature and specimens for related species, we found that this species did not match the known species of *Primula* and convinced us that this plant is novel and previously undescribed. Therefore, we describe and illustrate the plant as a new species here.

## ﻿Materials and methods

The morphological observation, measurements and description of the new species were based on both dried specimens and living plants from Yanbian County, Sichuan Province. Morphological comparisons with closely-related species were made by reviewing specimens from major herbaria, such as PE, P, E, KUN, SM and NY and relevant literature reports (Hu 1990; Hu and Kelso 1996; Xu et al. 2014a; Wang et al. 2022). The information regarding the type specimens involved in this study is as follows: *P.longipilosa*, Gengma TCM Resources Survey Exped. 5309260482, KUN1536784, KUN!; *P.neurocalyx* Franch., P.G. Farges 1369, P00649666, P!; *Primulasinomollis* Balf.f. & Forrest, Forrest, G. 5523, E00024096, E!. All morphological characters of *P.yanbianensis* and its morphologically similar species in the P.sect.Cortusoides, including *P.neurocalyx*, *P.longipilosa* and *P.sinomollis*, were measured using a Vernier caliper by living plants from their type localities . The conservation status of the new species was assessed following the guidelines of the IUCN Red List Categories and Criteria (IUCN Standards and Petitions Committee 2024).

## ﻿Taxonomic treatment

### ﻿ Primula yanbianensis

Taxon classificationPlantaeEricalesPrimulaceae

T.Shuai, Lei Cai & Z.K.Wu sp. nov.

A1D82914-CD78-5B10-A0D8-DE2B235516F6

urn:lsid:ipni.org:names: 77356677-1

#### Diagnosis.

**Figure 1.**
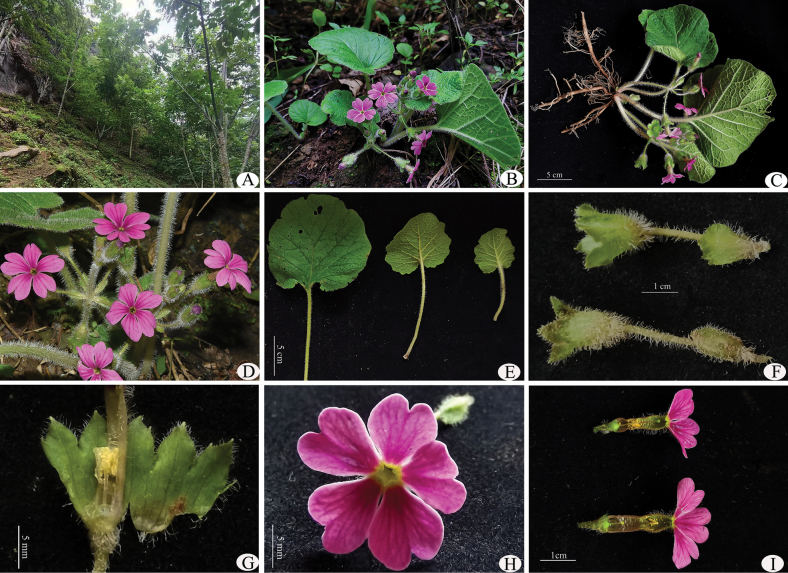
*Primulayanbianensis* sp. nov. **A** habitat **B** habit in flowering **C** fresh plant with roots **D** inflorescence **E** leaves, left: upper surface, right: lower surface **F** bracts and calyx **G** calyx and stamens **H** flower, front view **I** pin flower and thrum flower. Photographed by Z.K.Wu.

**Figure 2.**
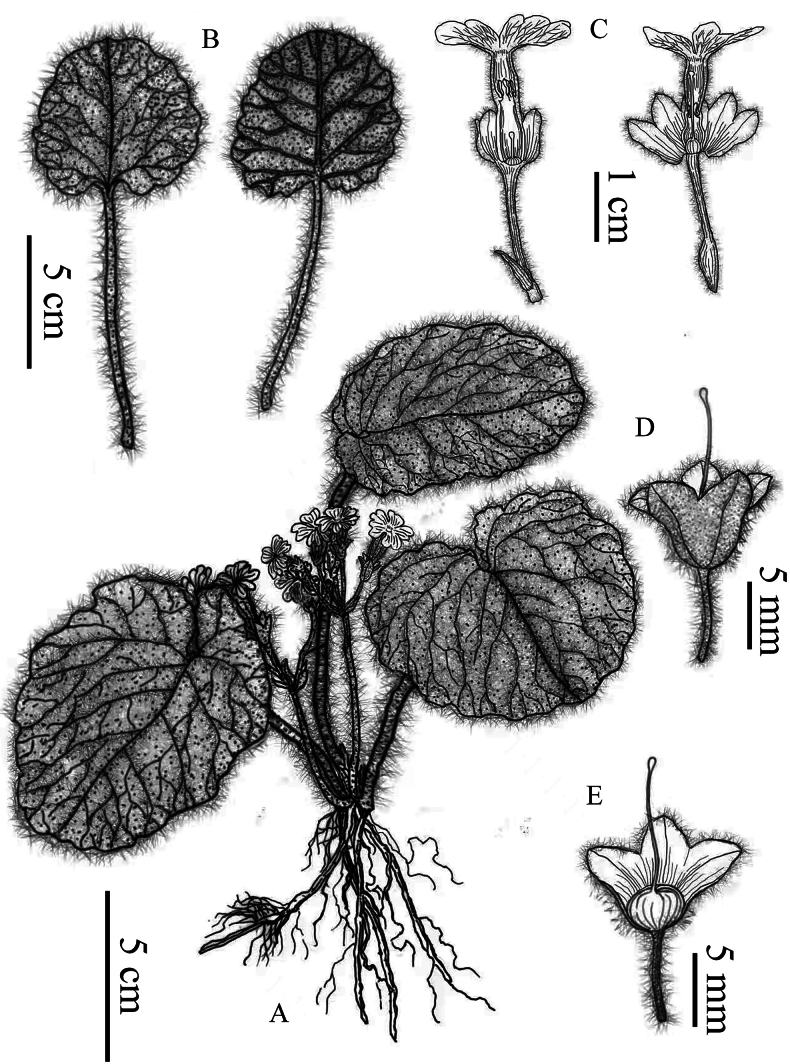
*Primulayanbianensis* sp. nov. **A** habit **B** leaves, left: upper surface, right: lower surface **C** flower, left: thrum flower, right: pin flower **D** calyx and stigma **E** calyx and ovary. Drawn by Ms. Xiang-Li Wu.

**Figure 3.**
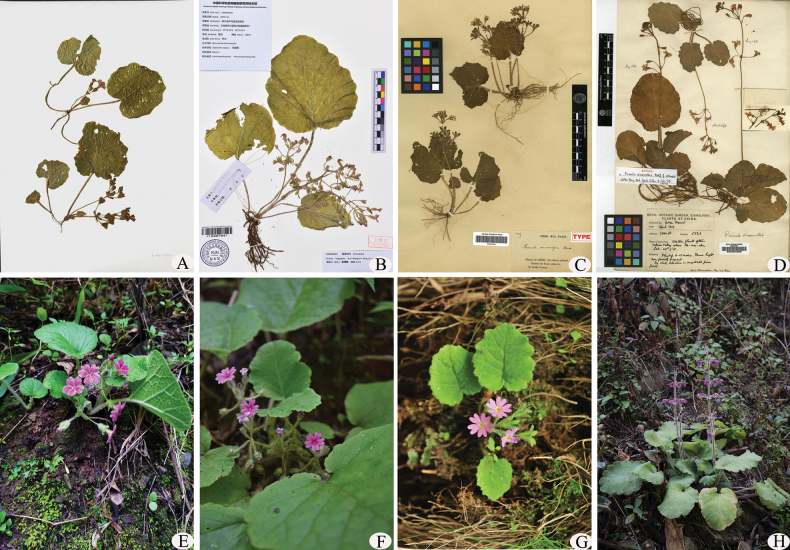
*Primulayanbianensis* and three of its allies **A** holotype of *P.yanbianensis* (L.Cai 2017070, KUN!) **B** holotype of *P.longipilosa* (Gengma TCM Resources Survey Exped. 5309260482, KUN!) **C** type of *P.neurocalyx* (P.G. Farges 1369, P!) **D** syntype of *P.sinomollis* (Forrest, G.5523, E!) **E***P.yanbianensis***F***P.longipilosa***G***P.neurocalyx***H***P.sinomollis*. **E, G, H** photographed by Z.K. Wu from their type locality, **F** photographed by Li Chen from its type locality.

The new species is most similar to *P.neurocalyx*, *P.longipilosa* and *P.sinomollis*, sharing multicellular hairs covering the plant, subrounded leaf blade are more or less lobed, with distinct petiole and a deeply cordate base and campanulate or narrowly campanulate calyx. However, the new species is distinguished from the latter three mainly by its scape usually being lower than leaf clusters, umbel is in whorls of 1–2, broadly ovate bracts and rose to pink petals are distinctly veined (Figs 1–3). The main morphological distinctions between *P.yanbianensis*, *P.neurocalyx*, *P.longipilosa* and *P.sinomollis* are summarised in Table 1.

**Table 1.** Comparison of main morphological characters amongst *P.yanbianensis*, *P.neurocalyx*, *P.longipilosa* and *P.sinomollis*.

Characters	*P.yanbianensis*	*P.longipilosa*	*P.neurocalyx*	*P.sinomollis*
Leaf blade	suborbicular to reniform, 3–10 cm in diameter, adaxially with sparsely multicellular hairs, abaxially with multicellular hairs along veins	cordate to broad cordate, 4–16 cm in diameter, covered with white soft multicellular hairs on both sides	orbicular or broadly ovoid, 3–7 cm in diameter, adaxially with sparsely multicellular hairs, abaxially with long hairs along veins	broadly elliptic to suborbicular, 3–8 cm in diameter, adaxially with appressed multicellular hairs, abaxially with long hairs along veins
Bract	broadly ovoid, 3–6 mm long	narrowly lanceolate, 5–15 mm long	linear-lanceolate, 6 mm long	linear to linear-lanceolate, 6–8 mm long
Scape	usually shorter than leaf clusters	shorter than or almost equal to leaf clusters	slightly longer than or equal to leaf cluster	usually much longer than leaf clusters
Inflorescence	umbels, 1–2 whorled, with each round containing 3–5 flowers	racemes mostly, sometimes umbels, 2, 3 rising from the rosette leaves, each scape has 7–25 flowers arranged in a racemose inforescence	umbels, 1, 2 whorled, with each round containing 3–7 flowers	umbels, 3–10 whorled, with each round containing 3–9 flowers
Calyx	6–8 mm long, lobes broadly ovate	6–10 mm long, lobes triangular to ovate-triangular	7–9 mm long, lobes rectangular to rectangular-lanceolate, herbaceous	5, 6.5 mm long, distinctly 5-ribbed, lobes narrowly lanceolate
Flowers	heterostylous, corolla rose to pink with distinctive veins on all part of lobes, yellow eye	heterostylous, corolla pink to pink rose, with veins prominent at base of lobes, inconspicuous at apex, purple eye	homostylous, corolla red-purple, lack veins on lobes, yellow eye	heterostylous, corolla light red to magenta or purple-red, lack veins on lobes, purple eye
Flowering time	June to July	July	May to July	April

#### Type.

China • Sichuan: Panzhihua City, Yanbian County, Yongxing Town. 27°4'32.95"N, 101°24'17.75"E, 1530 m alt., 28 June 2017 (fl.), *Zhikun WU & Lei Cai*, L.Cai2017070; 15 July 2020 (fl.), *Zhikun WU*, ZKWu2020053 (***holotype***: KUN!; ***Paratype***: KUN!).

#### Description.

A perennial herb with a short and usually inconspicuous rhizome and numerous robust fibrous roots, densely covered throughout with multicellular hairs. ***Leaves*** all rising from the root, forming a rosette, leaves including the petiole 10–25 cm long, petiole 5–18 cm, clothed with long spreading soft multicellular hairs, slightly sheathed at the base; leaf blade suborbicular to reniform, 4–12 cm long, 3–10 cm wide, apex obtuse, base cordate to deeply cordate, the leaf margin is undulate and lobed, the upper surface of the leaf sparsely septate hairs, while the lower surface covered with multicellular hairs along all the veins, mid-rib and 4, 5 pairs of pinnate lateral veins slightly impressed above, prominent below, reticulation open and rather feebly developed. ***Scapes*** 6–13 cm long, 1, 2 rising from the middle of the rosette leaves, shorter than leaves, densely covered with long soft multicellular hairs, carrying 1, 2 superposed umbels each with 3–5 flowers. ***Bracts*** broadly ovoid, 3–6 mm long, usually shorter than half of the pedicel, with long soft multicellular hairs. ***Pedicel*** 1, 2 cm, with a dense covering of long multicellular hairs. ***Flower*** heterostylous. ***Calyx*** narrowly campanulate to campanulate, 6–8 mm long, covered with long multicellular hairs abaxially, inner surface glabrous, cut to middle, lobes broadly ovate, with 3–5 prominent veins, apex with short cusp. ***Corolla*** rose to pink, tube 1.2–1.8 cm long, with long soft multicellular hairs outside, limb 1.5–2 cm in diameter, lobes obcordate, 6–8 mm in diameter, with prominent veins from yellow mouth, apex deeply emarginate. ***Pin flowers***: tube 1.2–1.5 cm long, style 10–12 mm long, stamens at ca. 3 mm above the base of corolla tube; ***thrum flowers***: tube 1.2–1.8 cm long, style 3–5 mm long, stamens at 2/3 length of corolla tube, ca. 10–12 mm above the base of corolla tube. Ovary globose (Figs 1, 2). ***Capsule*** unknown.

#### Distribution and habitat.

**Map 1.**
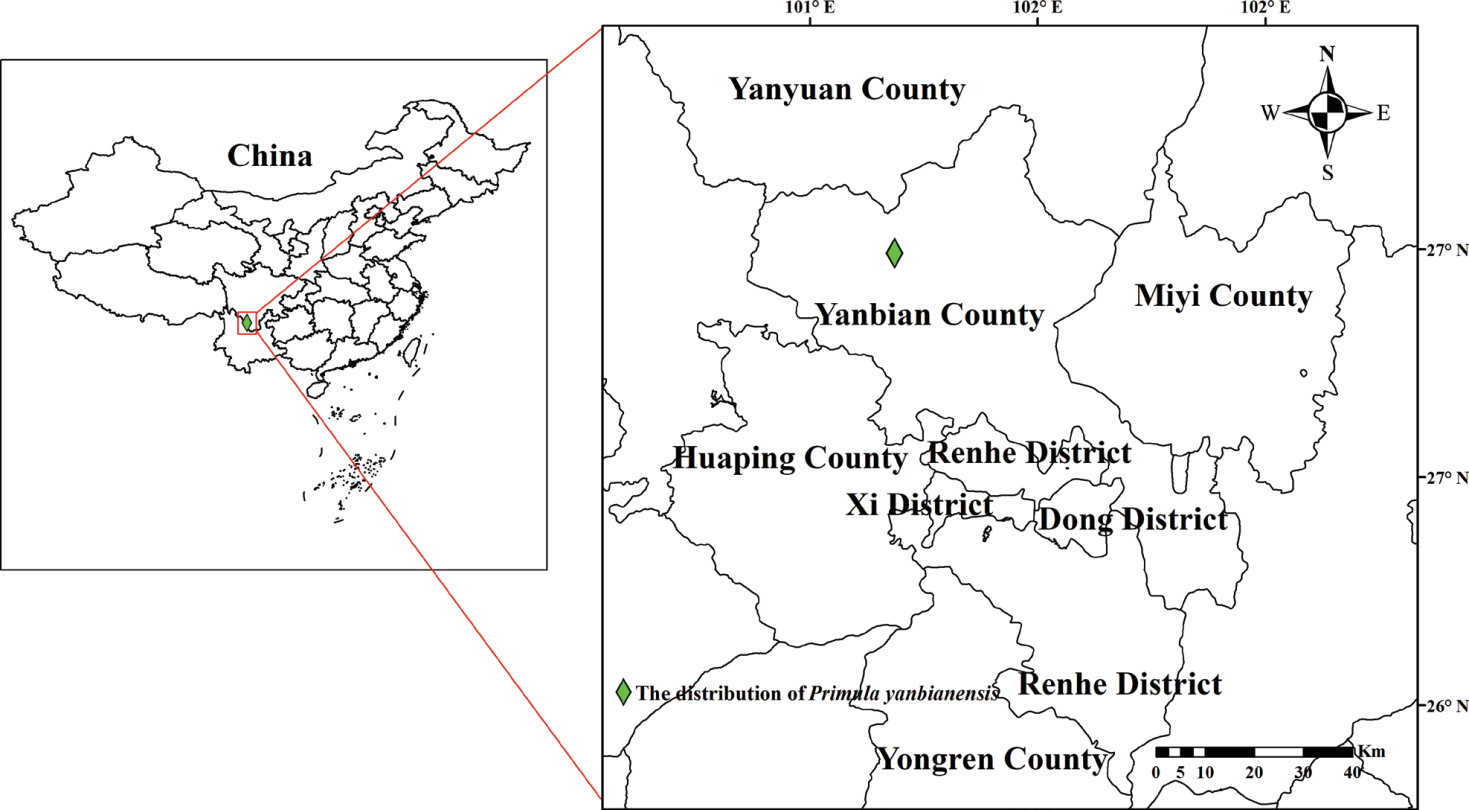
Location of the distribution of *Primulayanbianensis* in Yanbian County, Sichuan.

This new species is currently only known from the type locality near Qingyi Road, Yongxing Town, Yanbian County, Panzhihua City, Sichuan Province, China and is mostly found on grassy slopes along the valley forest margin, at altitudes of 1500–1650 m (Fig. 1; Map 1).

#### Phenology.

Flowering occurs from June to July.

#### Etymology.

The specific epithet of the new species is taken from the Chinese Pinyin “yanbian”, the name of the county in south-western Sichuan, China, where the type specimen was collected (Map 1).

#### Vernacular name.

Chinese mandarin: yan bian bao chun (盐边报春).

#### Conservation status.

This new species is a rare species with a restricted distribution. Currently, only one population with less than 150 individuals has been found in the type locality. We have observed a steady decline in the territory area of the habitat due to road construction and grazing, based on the latter two field expeditions conducted in 2020 and 2024. Therefore, in combination with the information currently available and in accordance with the guidelines for the use of the IUCN Red List Categories and Criteria (IUCN 2024), the conservation status of this new species has been assessed as ‘Critically Endangered’ (CR B1ab(i, iii)).

#### Additional specimens examined

**(*paratypes*).** • The same locality as holotype, 18 June 2024, *Tian Shuai*, TSh 2024045 (KUN!).

## ﻿Discussion

In the subgenus Auganthus of *Primula*, which encompasses sections such as *Auganthus*, *Monocarpicae*, *Cortusoides*, *Malvacea*, *Pycnoloba*, *Obconicolisteri*, *Reinii*, *Ranunculoides* and *Bullatae*, many species exhibit similar leaf characteristics. These include long petioles and multicellular hairs with leaf blades that are suborbicular to reniform in shape and deeply cordate at the base. Consequently, when encountering a plant within this subgenus, it is essential to determine the appropriate section for classification. In these closely-related sections, variations in calyx structure can be instrumental in distinguishing them from one another. Upon discovering *P.yanbianensis* in Yanbian, we considered whether it might belong to sect. Malvacea; however, its calyx traits are distinctly different from those found within the *Malvacea* series of *Primula*. The calyx characteristics of members belonging to sect. Malvacea serve as key diagnostic features that differentiate them from other sections: they possess foliaceous calyxes with more or less prominent well-developed reticulate venation and an obconical tube that expands into broad spreading lobes. During fruiting stages, the calyx enlarges to form a platter-like expansion at its center where a globose capsule rests; notably thickened throughout its entirety. Although *P.malvacea* is also present in southwestern Sichuan and adjacent northwestern Yunnan, which exhibiting significant morphological variation in inflorescences and flowers, and we observed both verticillate inflorescence types and racemose inflorescence types within this region as well. Nevertheless, the foliaceous calyx traits of *P.malvacea* are markedly distinct from the campanulate calyx characteristic of *P.yanbianensis* (Fig. 1D). This distinction is why we did not classify this new species under sect. Malvacea; instead based on its campanulate calyx morphology we have classified *P.yanbianensis* within sect. Cortusoides.

Morphologically, P.sect.Cortusoides is characterised by a lack of farina, the plants are generally covered with multicellular hairs, the petiole is strongly conspicuous, the leaf blades are suborbicular to reniform, the margins are lobed, the bases are cordate to deeply cordate and the calyxes are campanulate or narrowly campanulate (Smith and Fletcher 1944). These traits make it easy to determine at the sectional level. However, some traits are overlapping in the closely-related species in this section, making it difficult to identify these species in field, for example, *Primulamollis* group (*P.mollis* Nutt.ex Hook., *P.sinomollis*, *P.neurocalyx* and *P.longipilosa*), *Primulavaginata* group (*P.vaginata* Watt, *Primulaseptemloba* Franch., *Primulapalmata* Hand.-Mazz. and *Primulaloeseneri* Kitag.), as well as *Primulacinerascens* group (*Primulacinerascens* Franch., *Primulapolyneura* Franch., *Primulascopulicola* G.Hao, C.M.Hu & Y.Xu and *Primulasieboldii* E.Morren). The new species *P.yanbianensis* is similar to *P.longipilosa*, *P.neurocalyx* and *P.sinomollis*, but *P.yanbianensis* can be easily separated from these three species by its umbel being in whorls of 1–2, scapes usually lower than the leaf clusters, bracts broadly ovate and rose to pink petals with conspicuous veins. Overall, the new species is most closely related to *P.longipilosa* morphologically, but *P.longipilosa* is mainly distributed in Yunnan near the border to Myanmar (Wang et al. 2022), whereas the new species is distributed in Sichuan and no similar populations have been found in the intermediate area between these two. Therefore, *P.yanbianensis* might have diverged from their ancestors after geographical isolation, but its phylogenetic relationships need further investigation.

## Supplementary Material

XML Treatment for Primula yanbianensis

## ﻿Additional information

### Author ORCIDs

Tian Shuai https://orcid.org/0009-0009-7314-8026

Hong-qiang Lin https://orcid.org/0000-0001-8152-4911

Lei Cai https://orcid.org/0000-0002-9251-2745

Yu-fan Chen https://orcid.org/0009-0001-8086-4444

Zhi-Kun Wu https://orcid.org/0000-0002-4583-7782

### Data availability

All of the data that support the findings of this study are available in the main text.
